# Supplementary material for: Spatial Variation in Stream-Water Bacterial Communities Across Sites Differing in Wild Chinese Giant Salamander Detection Frequency
Source: Biology (Basel). 2026 Sep 18;15(18):1647. doi: 10.3390/biology15181647 (PMC13604818; doi:10.3390/biology15181647)
Supplement: Supplementary file 1 [file biology-15-01647-s001.zip › Data Related to “Spatial Variation in Stream-Water Bacterial Communities across Sites Differing in Wild Chinese Giant Salamander Detection Frequency”/Microbial Data/result/05.BetaDiversity/beta_pcoa/sample/weighted_unifrac/PCoA_circleLabel.pdf]

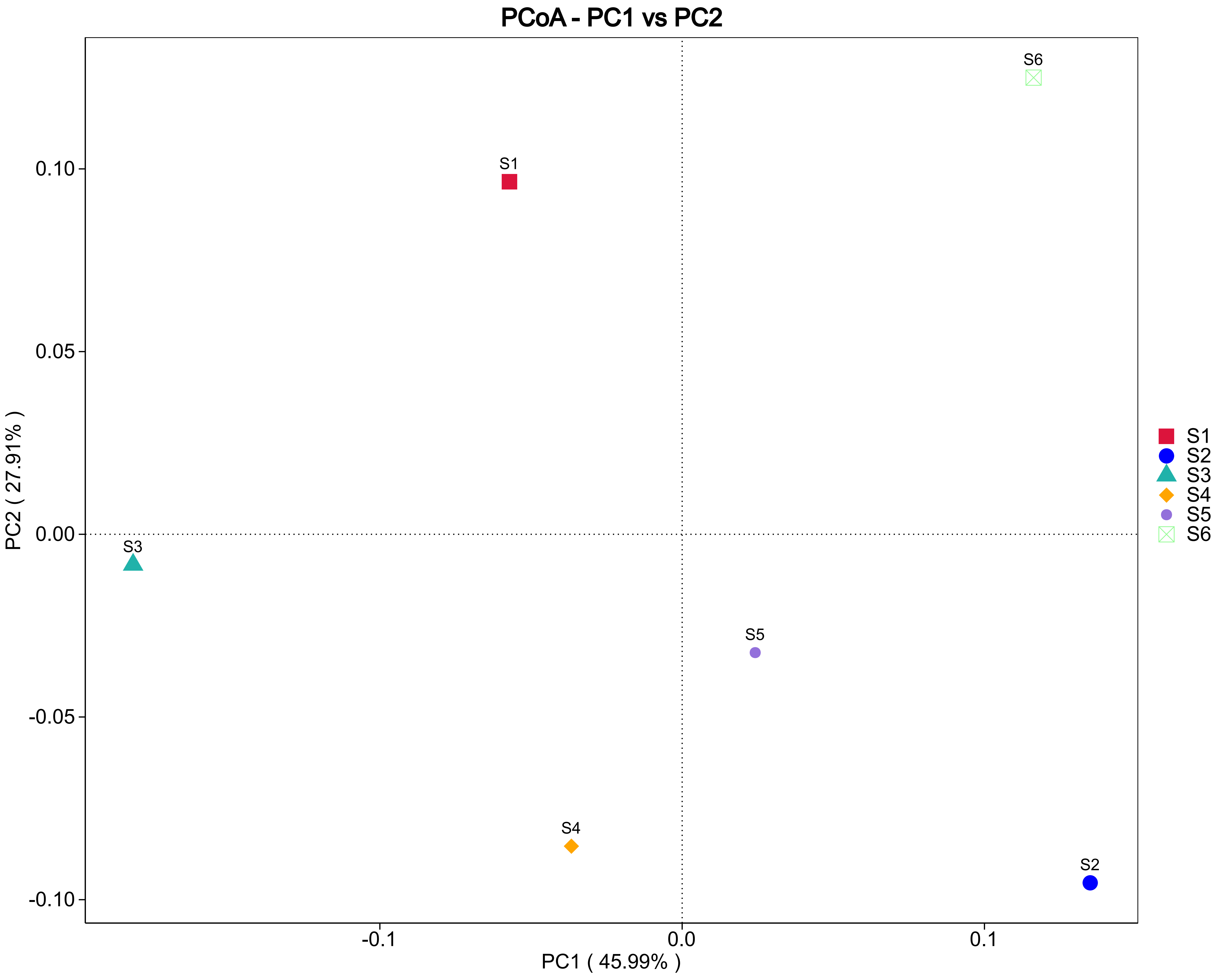

PCoA - PC1 vs PC2
PC2 (27.91%)
PC1 (45.99%)
0.10
0.05
0.00
-0.05
-0.10
-0.1
0.0
0.1
S1
S2
S3
S4
S5
S6
